# Comparison of Fundus Autofluorescence and Indocyanine Green Angiography in Multiple Evanescent White Dot Syndrome

**DOI:** 10.1016/j.xops.2025.100731

**Published:** 2025-02-04

**Authors:** Valentine Labattut, Yasmine Serrar, Armelle Cahuzac, Pierre Gascon, Martine Mauget-Faÿsse, Benjamin Wolff, Mariam Ghazaryan, Pascal Seve, Laurent Kodjikian, Thibaud Mathis

**Affiliations:** 1 Service d'Ophtalmologie, Hôpital universitaire de la Croix-Rousse, Hospices Civils de Lyon, Lyon, France; 2 Fondation Ophtalmologique Adolphe de Rothschild, Paris, France; 3 Groupe Almaviva Santé, Clinique Juge, Marseille, France; 4 Centre Ophtalmologique Maison Rouge, Strasbourg, France; 5 Teona Ophthalmology Clinic, Yerevan, Armenia; 6 Service de Médecine Interne, Hôpital universitaire de la Croix-Rousse, Hospices Civils de Lyon, Lyon, France; 7 Laboratoire UMR-CNRS 5510 Matéis, Villeurbanne, France

**Keywords:** Multiple evanescent white dot syndrome, MEWDS, Physiopathology, Uveitis

## Abstract

**Purpose:**

The aim of this study was to compare fundus blue autofluorescence (BAF) images, indicating photoreceptor alteration, and indocyanine green angiography (ICGA), indicating retinal pigment epithelium (RPE) alteration, in multiple evanescent white dot syndrome (MEWDS) to investigate the initial damage location within the RPE-photoreceptor complex.

**Design:**

Multicentric retrospective cohort study carried out across tertiary centers for retinal and inflammatory diseases in France.

**Participants:**

A total of 31 eyes affected by primary MEWDS were included.

**Methods:**

Only primary MEWDS, with sufficiently high-quality images, were included, and their data were analyzed cross-sectionally at baseline and at the recovery phase, between 4 and 8 weeks. A standardized protocol was set up for measuring the areas affected by MEWDS on the late-phase ICGA and on BAF; this was always carried out by the same investigator. On 55° macular-centered images, both the BAF and ICGA areas were delimited and calculated in the FIJI/ImageJ Software and then compared with each other. The same process was used to compare macular areas bounded by a 6-mm diameter Early Treatment Diabetic Retinopathy Study circle centered on the fovea.

**Main Outcome Measures:**

Areas in mm^2^ affected by MEWDS on BAF and ICGA.

**Results:**

The median hypofluorescent area on ICGA (93.03 mm^2^, interquartile range [IQR: 54.08–134.00]) was significantly larger than that affected on BAF (76.22 mm^2^ [IQR: 43.65–122.20], *P* < 0.0001). The median damaged surface was 37.21% (IQR: 21.63%–53.61%) on ICGA vs. 30.49% (IQR: 17.15%–46.60%) on BAF (*P* < 0.0001). Regarding only the macular area, damage was also significantly larger on ICGA than in BAF (15.16 [9.55–24.08] mm^2^ vs. 13.48 [4.87–17.82] mm^2^, *P* < 0.0001).

**Conclusions:**

Our study showed that MEWDS lesions are more extensive in ICGA than in BAF, indicating a predominant RPE dysfunction. We therefore support the hypothesis that MEWDS is a primary retinal pigment epitheliopathy, causing reversible and nondestructive dysfunction of the RPE and photoreceptors. Our results support recent analysis of modern multimodal imaging. Further studies using en face OCT are needed to analyze the outer retinal layers and corroborate the hypothesis.

**Financial Disclosure(s):**

The author(s) have no proprietary or commercial interest in any materials discussed in this article.

Multiple evanescent white dot syndrome (MEWDS) is a rare, retinal inflammatory disease characterized by white dots localized on the posterior pole and mid-periphery.^1^ Multiple evanescent white dot syndrome is usually a unilateral disease^2^ that typically affects younger women with mild myopia and resolves on its own within a few weeks.^1^ It can either be idiopathic or triggered by a chorioretinal disease.^3^, ^4^, ^5^ Two types of lesions are well characterized in spectral domain OCT (SD-OCT): “spots,” which are disruption of the ellipsoid zone, and “dots,” which are hyperreflective spicules in the outer nuclear layer.^6^^,^^7^ Although the physiopathology of MEWDS is still uncertain, many advancements have been made since its first description in 1984 by Jampol et al.^1^ Hypofluorescence on late-phase indocyanine green angiography (ICGA) was initially described as a sign of choroidal hypoperfusion.^8^ However, in 2008, it was suggested that it may be due to damage to the retinal pigment epithelium (RPE)-photoreceptor complex.^9^ This hypothesis was confirmed by multimodal imaging, particularly OCT angiography, which showed that the choriocapillaris remained intact.^10^ Multiple evanescent white dot syndrome, hence, could be a “photoreceptoritis” with secondary RPE involvement^10^ rather than a choriocapillaritis since the absence of hypoperfusion of the choriocapillaris on OCT angiography has been demonstrated several times.^11^, ^12^, ^13^, ^14^, ^15^ It is now assumed that dark spots on late-phase ICGA are due to a deficit in the internalization of indocyanine green (ICG) molecules coming from the choroid by the RPE.^15^, ^16^, ^17^ Moreover, MEWDS lesions are hyperautofluorescent in blue autofluorescence (BAF), disappearing after photobleaching.^18^^,^^19^ This specific pattern on the autofluorescence image suggests an unmasking of normal RPE fluorescence secondary to the loss or misalignment of the outer segments of the photoreceptors.^10^^,^^17^^,^^19^^,^^20^ Although it has been shown that MEWDS is a disease of the RPE and the photoreceptors, there is still controversy about the exact physiopathology.^11^^,^^12^^,^^14^ It is difficult to determine whether MEWDS affects the RPE or the photoreceptors first. In our study, we propose to compare the area of MEWDS lesions on ICGA, indicating damage to the RPE, with the area of MEWDS lesions on BAF, indicating damage to the photoreceptors. The aim is to provide an additional argument for the initial location of the damage within the RPE-photoreceptor complex.

## Methods

### Inclusion Criteria

We retrospectively recruited patients who presented with MEWDS between July 2010 and August 2024 in 3 references centers for retinal and inflammatory diseases in France (Hospices Civils de Lyon-Lyon, Fondation Adolphe de Rothschild-Paris, and Centre Monticelli-Marseille). We specifically chose the start date of the study following harmonization in our reference centers of a common protocol for performing BAF and angiography sequences.

The diagnosis of MEWDS was established, according to the Standardization of Uveitis Nomenclature criteria, by ≥2 experts in retinal pathology. As we previously reported, we defined 2 forms of MEWDS: the idiopathic form of MEWDS, which we called “primary MEWDS,” and MEWDS associated with underlying chorioretinal disease, which we referred to as “secondary MEWDS.”^4^ In the present study, we exclusively recruited patients with primary MEWDS.

Therefore, patients not meeting diagnostic criteria, those with trigger-MEWDS, and those with poor-quality imaging unsuitable for accurate analysis were excluded. This research was conducted in accordance with the Declaration of Helsinki. Informed consent was obtained for the human subjects in this study (Ethics Committee of the French Society of Ophthalmology, IRB 00008855 Société Française d'Ophtalmologie IRB#1).

### Data Collection

All data were collected cross-sectionally from the medical records at baseline during the acute phase (T0), up to 4 weeks after the onset of symptoms and within a timeframe spanning from 4 to 8 weeks postsymptom onset (T1), commonly referred to as the recovery phase.

Clinical data included age, sex, spherical equivalent, presence or absence of flu-like symptoms, best-corrected visual acuity (BCVA, logarithm of the minimum angle of resolution [logMAR]), slit-lamp examination, and dilated fundus examination. For multimodal imaging, retinographies were taken using either the Eidon retinograph (CenterVue) or the ultra-widefield Optos California system (Optos PLC). Spectral domain OCT was performed on the Spectralis SD-OCT. The same applies to the BAF, fluorescein angiography (FA), and ICGA images produced on the Spectralis HRA (Heidelberg Engineering).

### Inflammation Scores

We evaluated inflammation using 2 scores of 3 points each, measured at T0 and T1.

1. A clinical score of 0 to 3: 1 point for the presence of Tyndall effect, 1 point for the presence of vitritis, and 1 point for optic nerve edema.
2. An imaging score of 0 to 3: 1 point for papillitis on FA, 1 point for vasculitis on FA, and 1 point for vitritis on SD-OCT.

### Total Areas on ICGA and BAF Analysis

In our study, we calculated and compared the areas affected by MEWDS lesions on the late phase of ICGA and on BAF (excitation wavelength: 488 nm) for each patient and performed on the same imaging device. Because BAF images are known to be sometimes artifacted or lack reproducibility,^21^ we only selected images in which the lesions had sufficient contrast and were centered on the posterior pole. Most importantly, we implemented a strict protocol to only analyze lesions that were common to both ICGA and BAF images. To ensure comparability of images and reproducibility of measurements, we developed a standardized protocol, always carried out by the same investigator (V.L.). Initially, the areas were measured for each eye at baseline (T0) on 55° macula-centered images using Spectralis HRA. Both BAF and ICGA images were acquired on the same day. Indocyanine green angiography images were captured at a late phase between 20 and 35 minutes. All images were adjusted to the same dimensions of 1536 × 1536 pixels and the same resolution of 87.5 pixels per inch. The ICGA and BAF images of each patient were loaded into the GIMP software (version: 2.10.36, free and open-source software). The images were then overlaid and adjusted using the layer module so that all structures were perfectly aligned. Any areas that were not common to both images were masked. Then we used the freehand tool in the FIJI/ImageJ software (version: 2.14.0/1.54f, ImageJ) to delineate the borders of hypofluorescent and hyperfluorescent lesions in ICGA and BAF, respectively. Before any measurements, the software was calibrated using a scale bar of 200 μm for all images. Because the optic nerve is hypoautofluorescent, its border was drawn with the freehand tool and then removed from the hypofluorescent total area in ICGA. Similarly, it is recognized that the foveola exhibits inherent hypoautofluorescence primarily attributable to the presence of carotenoid pigments like lutein and zeaxanthin, which possess a strong affinity for absorbing the blue light utilized in autofluorescent imaging. Furthermore, lipofuscin is less prevalent in this region of the RPE. Consequently, the elliptical tool was employed to delineate a circle encompassing the area of hypoautofluorescence in the fovea. The same area was then subtracted from the total hypo-ICGA area. All these areas were recorded in the region of interest manager to calculate the area of MEWDS lesions in mm^2^. Finally, we divided the affected area by the total area of each image, which is 250 mm^2^ after calibration of all images, to obtain the percentage of area affected in both ICGA and BAF.

### Macular Areas on ICGA and BAF

Using the elliptical tool on the calibrated images mentioned above, we created a 6-mm diameter circle centered on the fovea that corresponds to the widest circle of the Early Treatment Diabetic Retinopathy Study macular grid. This circle represented a standardized macular area of 28.27 mm^2^ ($A=\pi r^{2}$) for each patient. We then overlaid this macular area on the total area of MEWDS lesions and selected the common area between these 2 in order to calculate the area of macular MEWDS lesions.

### Visual Acuity Analysis

At T0, we divided our study population into 2 groups depending on their initial visual acuity (VA): 1 group for VA <0.2 logMAR and second one for VA ≥0.2 logMAR. We then compared the initial VA with the macular damage calculated as explained previously. At T1, a study of the correlation between visual recovery and the extent of lesions was carried out.

### Statistics

Categorical variables are expressed as count and percentage, and continuous variables are expressed as median and interquartile range (IQR). Comparisons between paired data were performed using the Wilcoxon matched-pairs signed rank test. For continuous variables, the comparisons between the groups were calculated using the nonparametric Mann–Whitney test according to the normality. The correlation between VA and pathological macular area was assessed using the Spearman's correlation coefficient r. We also performed a multivariate analysis based on sex, age, and BCVA (<0.2 logMAR vs. ≥0.2 logMAR). Given the small sample size and the absence of gender parity, a nonparametric linear regression was applied to model the relationship between the dependent variables (ICGA and BAF) and the following predictors: sex, age, and baseline BCVA. Permutation tests were conducted to compute *P* values for the regression coefficients. This approach provides more reliable *P* values, especially for small samples. A *P* value <0.05 was considered statistically significant. All statistical analyses were performed using GraphPad Prism, version 10.2.3 (GraphPad Software).

## Results

### Study Population and Patient Characteristics at T0

A total of 103 eyes (102 patients) were diagnosed with MEWDS during the study period. Among them, 31 eyes on 30 patients had primary MEWDS and images of sufficiently good quality to be analyzed. The median age of patients was 25.50 years (IQR: 22.00–35.25), 28 (93.33%) were women, and the median spherical equivalent was −0.75 diopters (IQR: 1.25–0.25). Only 1 male patient (3.33%) had bilateral MEWDS, and 4 patients (13.33%) presented a history of flu-like symptoms. At T0, all patients complained of visual symptoms with a median BCVA of 0.2 logMAR (IQR: 0.6–0.0) (Table 1).

**Table 1:** Patients Characteristics and Clinical Examination at T0

Characteristics	Eyes (n = 31)
Females, n (%)	28 (93.3)
Age, yrs, median (IQR)	25.50 (22.0–35.25)
Spherical equivalent, diopters, median (IQR)	−0.75 (−1.25 to 0.25)
Median BCVA, logMAR (IQR)	0.2 (0.4–0.0)
History of flu-like symptoms, n (%)	4 (13.3)
Visible spots/dots on fundus, n (%)	30 (96.8)
Clinical inflammatory signs, n (%)	
0	13 (41.9)
1	12 (38.7)
2	4 (12.9)
3	1 (3.2)
No data	1 (3.2)
Multimodal imaging inflammatory signs, n (%)	
0	0 (0)
1	8 (25.8)
2	14 (45.2)
3	8 (25.8)

BCVA = best-corrected visual acuity; IQR = interquartile range; logMAR = logarithm of the minimum angle of resolution.

All patients had received a multimodal imaging including at least ICGA, FA, BAF, and SD-OCT. Every case presented typical MEWDS lesions with hypofluorescence on ICGA, hyperautofluorescence on BAF, spicules on SD-OCT, and hyperfluorescent lesions on FA.

### Comparison between ICGA and BAF Total Areas

To ensure that ICGA and BAF are comparable on normal structure, we first measured the area of the optic disc, which was not significantly different on both imaging modalities, ICGA and BAF (3.17 [IQR: 2.76–3.57] mm^2^ vs. 3.20 [IQR: 2.79–3.56] mm^2^, respectively, *P* = 0.627).

**Figure 1:**
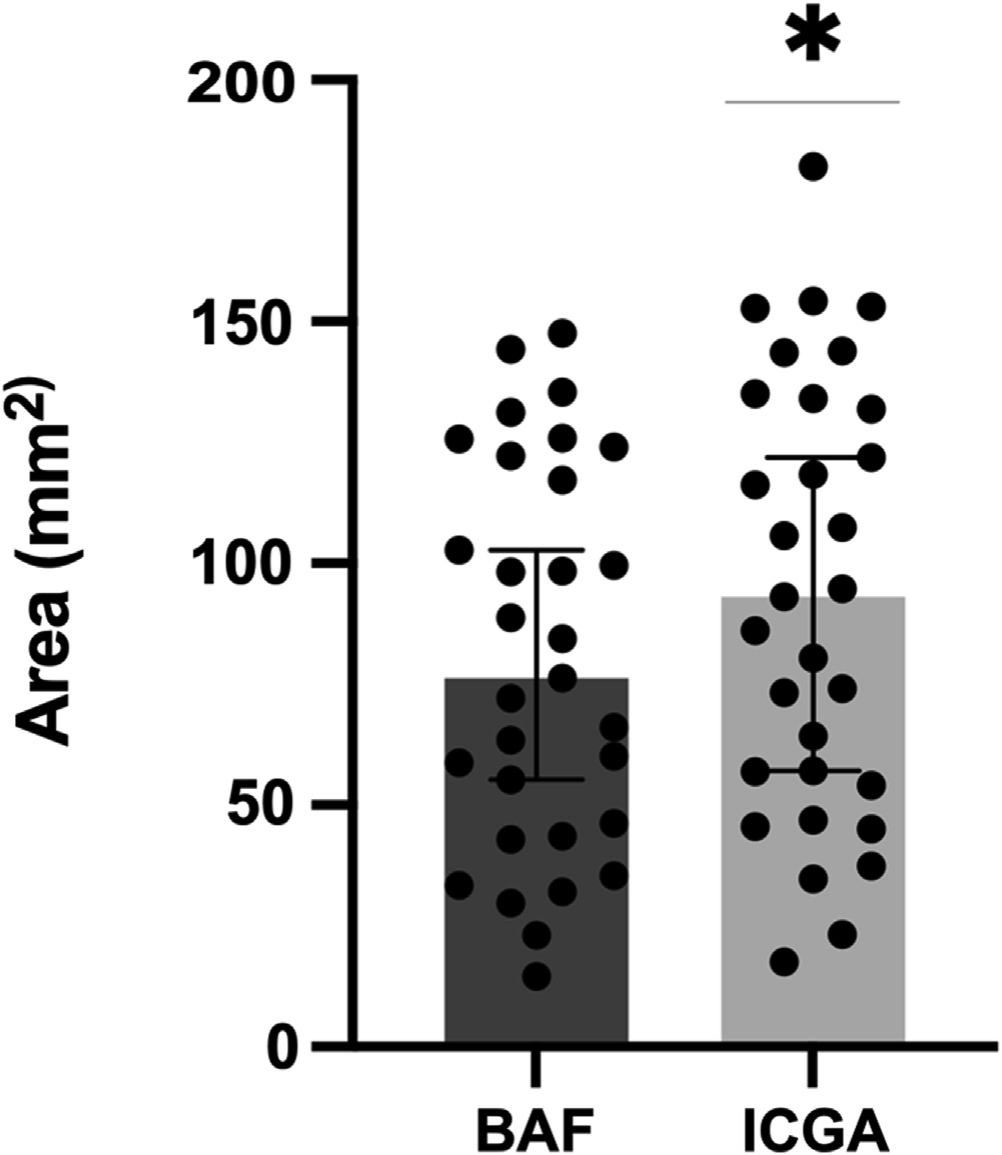
Median area (IQR) of MEWDS on the posterior pole, excluding the fovea. ∗*P* < 0.0001. BAF = blue autofluorescence; ICGA = indocyanine green angiography; IQR = interquartile range; MEWDS = multiple evanescent white dot syndrome.

**Figure 2:**
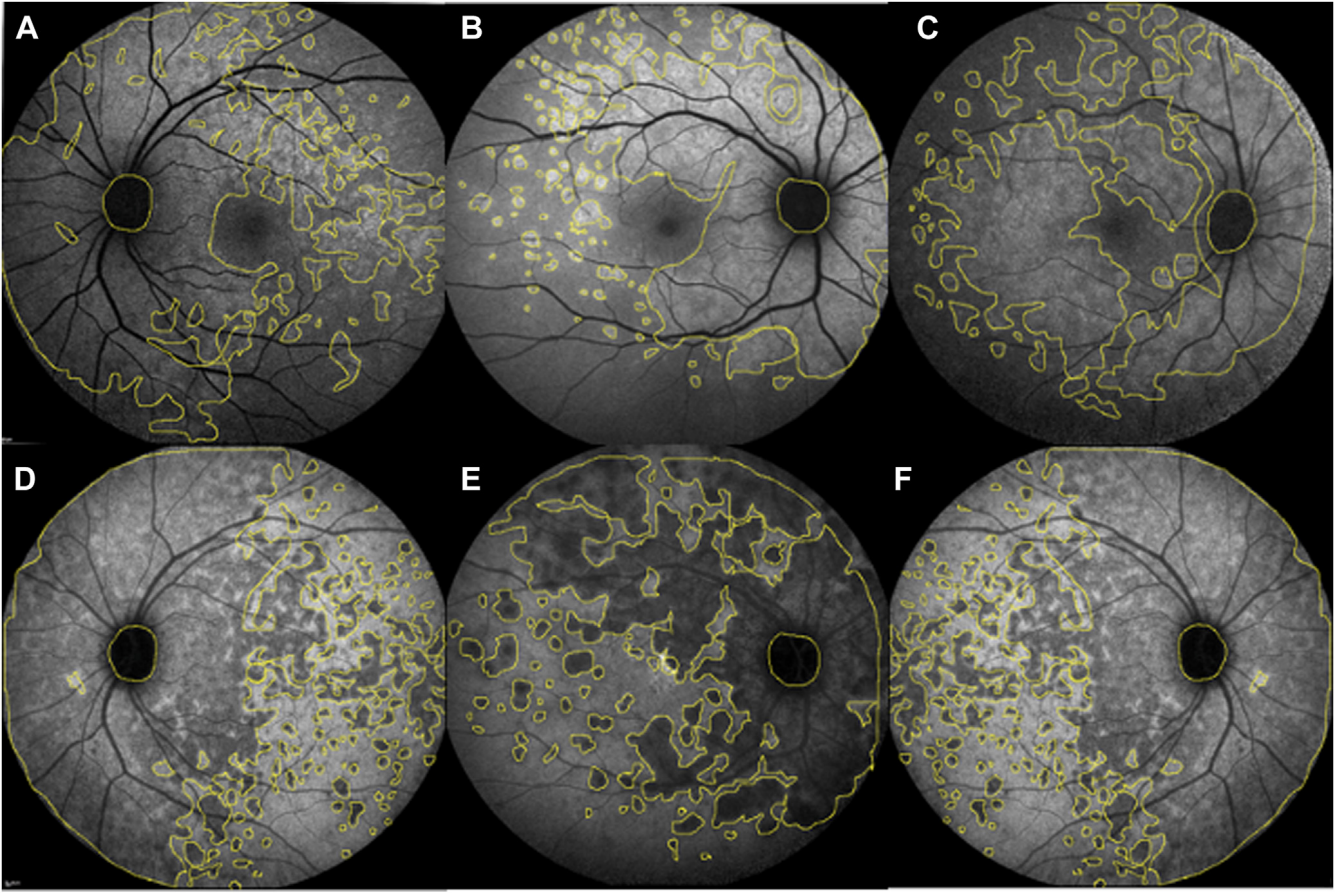
Three cases of ICGA hypofluorescence (**A–F**) and BAF hyperautofluorescence (**A**–**C**) measurement of MEWDS. BAF = blue autofluorescence; ICGA = indocyanine green angiography; MEWDS = multiple evanescent white dot syndrome.

After exclusion of the foveola area (mean = 0.34 mm^2^), the area of MEWDS lesions on late-phase ICGA was significantly larger than that on BAF (*P* < 0.0001). The median pathological area represented 37.21% (IQR: 21.63%–53.61%) of the ICGA's total surface area of 250 mm^2^ compared with 30.49% (IQR: 17.15%–46.60%) in BAF (*P* < 0.0001) (Table 2, Figs 1 and 2).

**Table 2:** Spatial Distribution of MEWDS Lesions at T0

	LP-ICGA n = 31	BAF, n = 31	*P* Value
Total area			
Median area of MEWDS lesions on the posterior pole excluding the fovea, mm^2^ (IQR)	93.03 (54.08–134.00)	76.22 (43.65–122.20)	<0.0001
Median pathological area, % (IQR)	37.21 (21.63–53.61)	30.49 (17.15–46.60)	<0.0001
Macular area			
Median area of MEWDS lesions on the macular area, mm^2^ (IQR)	15.16 (9.55–24.08)	13.48 (4.87–17.82)	<0.0001
Median macular pathological area, % (IQR)	53.63 (33.78–85.18)	47.68 (17.22–63.14)	<0.0001

BAF = blue autofluorescence; IQR = interquartile range; LP-ICGA = late-phase indocyanine green angiography; MEWDS = multiple evanescent white dot syndrome.

### Comparison between ICGA and BAF Macular Areas

In a 6-mm circle centered on the fovea, the macular pathological area was significantly larger in the late phase of ICGA than in BAF (*P* < 0.0001).

**Figure 3:**
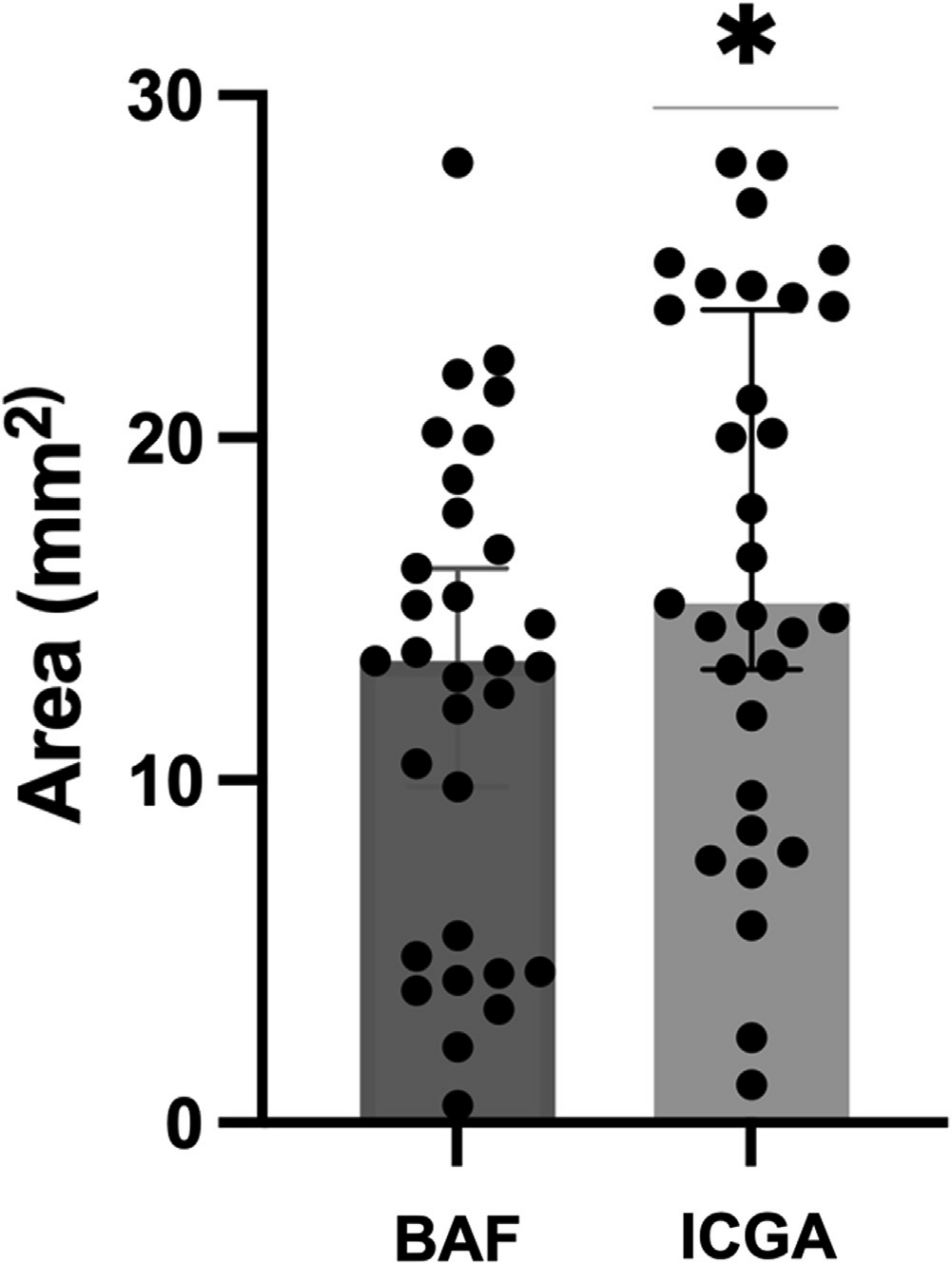
Median area (IQR) of MEWDS on the macula area, excluding the fovea. ∗*P* < 0.0001. BAF = blue autofluorescence; ICGA = indocyanine green angiography; IQR = interquartile range; MEWDS = multiple evanescent white dot syndrome.

**Figure 4:**
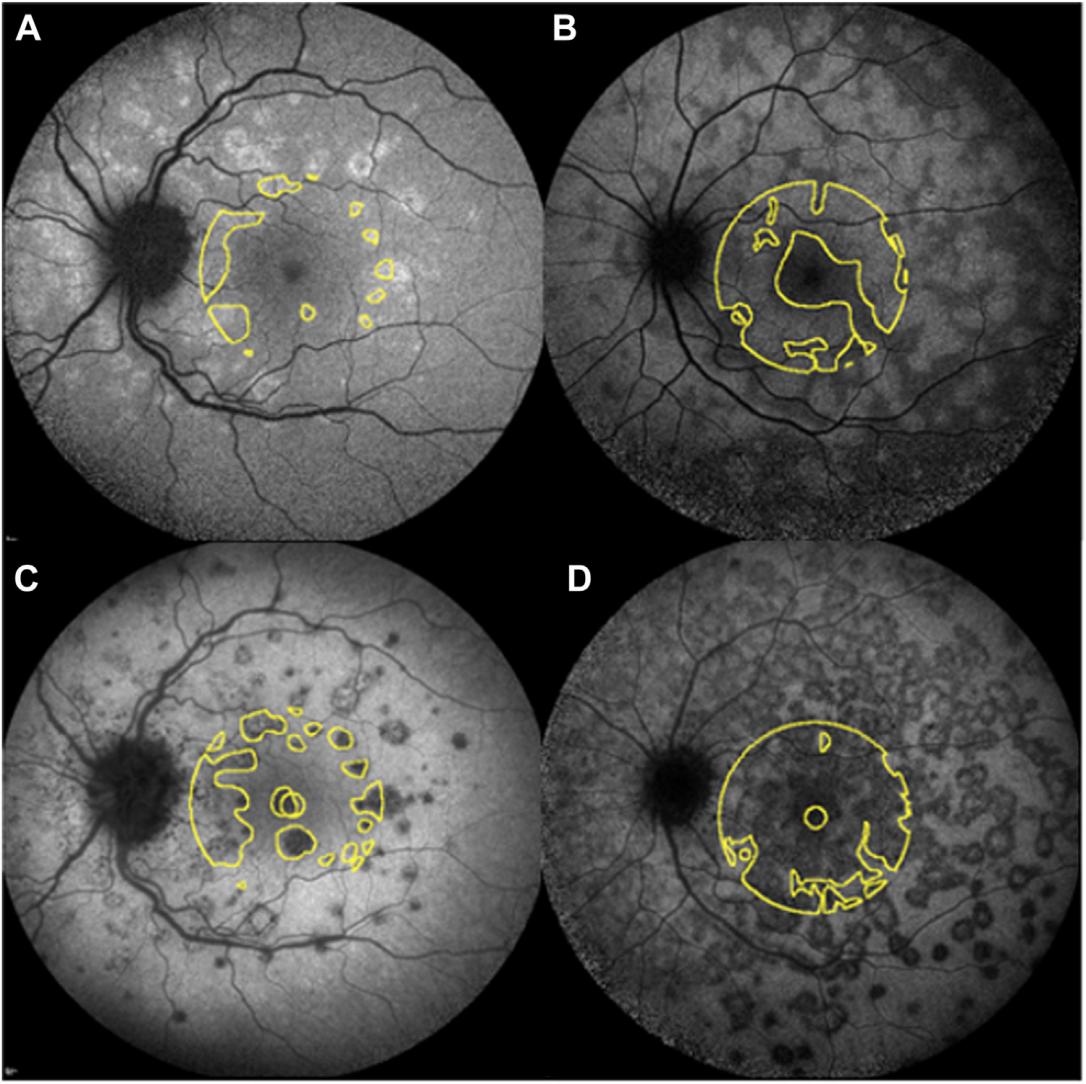
Cases of macular ICGA hypofluorescence (**C,D**) and BAF hyperautofluorescence (**A,B**) measurement of MEWDS. BAF = blue autofluorescence; ICGA = indocyanine green angiography; MEWDS = multiple evanescent white dot syndrome.

Then, we used these macular areas to look for a link between the initial loss of VA (at T0) and the extent of macular damage. To achieve this, we defined 2 groups of patients: those with VA <0.2 logMAR (n = 13) and those with a VA ≥0.2 logMAR (n = 15), compared according to their macular damage. There was no significant difference in macular damage between the low VA group and the higher VA group in either ICGA (14.49 [IQR: 8.22–24.78] mm^2^ vs. 16.51 [IQR: 11.89–23.84] mm^2^, *P* = 0.964) or BAF (13.49 [IQR: 4.27–28.03] mm^2^ vs. 13.30 [9.81–15.35] mm^2^, *P* = 0.555) (Table 2, Figs 3 and 4).

### Spectral Domain OCT and En Face OCT

**Figure 5:**
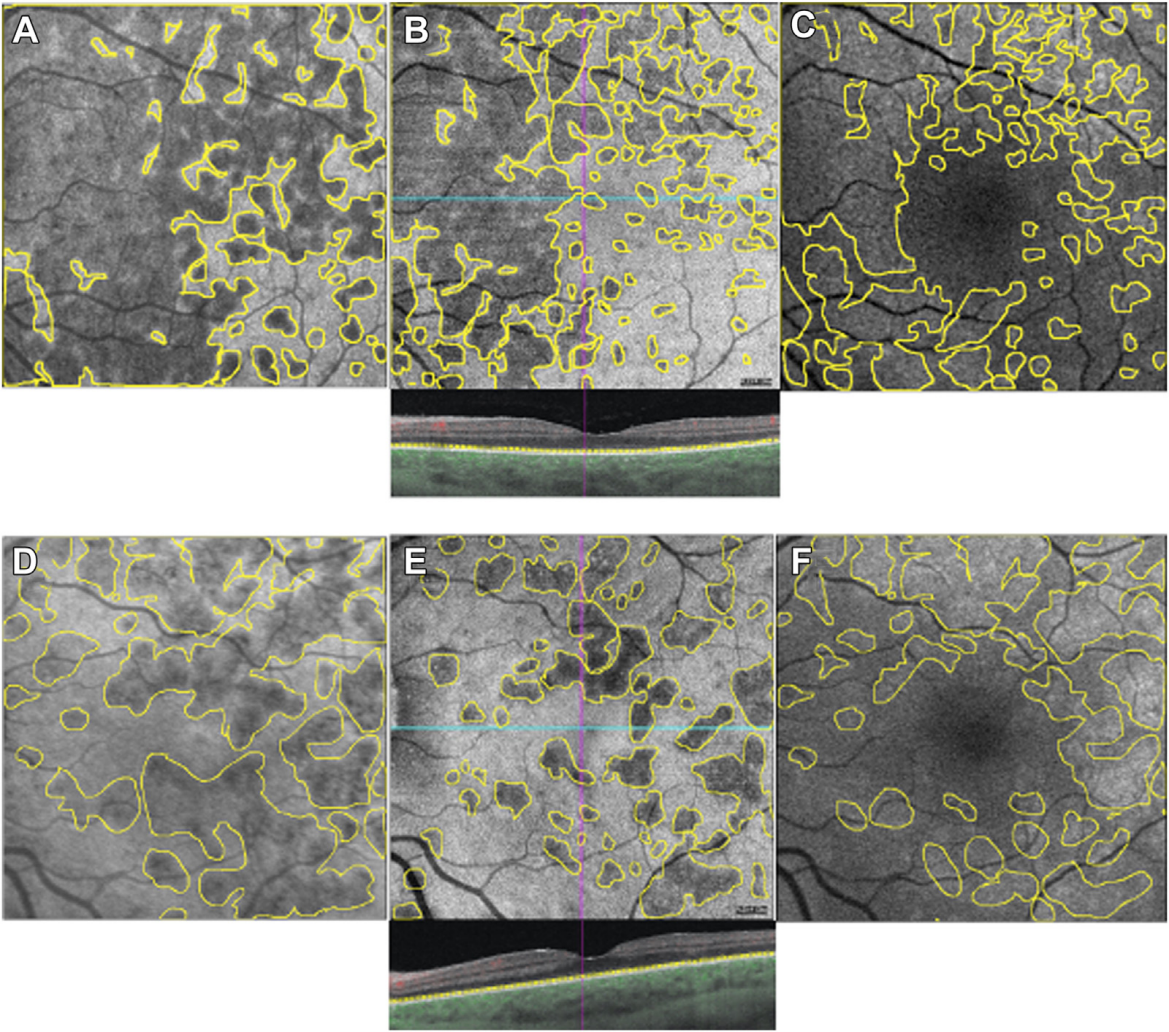
Comparison of indocyanine green angiography (ICGA; **A, D**), en face OCT (**B, E**), and blue autofluorescence (BAF; **C, F**) of 2 patients with MEWDS lesions. Note that ICGA shows a wider lesion area than en face OCT and BAF. MEWDS = multiple evanescent white dot syndrome.

At baseline, SD-OCT was performed on all patients and showed multiple areas of ellipsoid zone disruption that had the same general location as hypofluorescent lesions on ICGA and hyperautofluorescent lesions on BAF. Only 2 of our patients had an en face OCT, which allows a more detailed analysis of the outer layers of the retina and a precise comparison with BAF and ICGA. In these 2 cases, we noted that the alterations were located in the ellipsoid zone. They seemed smaller than the lesions seen on ICGA and overlapped better with the BAF images (Fig 5). Because of the limited number of images available, it was not possible to carry out a quantitative analysis.

### Recovery and Evolution of Multiple Evanescent White Dot Syndrome

Nineteen patients were seen for follow-up consultations at T1 (Table 3), with a median consultation time of 4.00 (IQR: 4.00–6.00) weeks. The overall clinical progression was positive, with a median BCVA of 0.00 (IQR: 0.00–0.00) logMAR at T1. There was no longer any clinical inflammation, although inflammation persisted on multimodal imaging in 2 cases, and the yellowish spots had faded but were still visible in 6 patients (31.58%). Finally, we found no correlation between the extent of lesions on the total area at the initial phase and final VA either in ICGA (r = −0.020, *P* = 0.934) or in BAF (r = 0.032, *P* = 0.896). We obtained the same result using macular area, with a negative correlation coefficient on both ICGA (r = −0.287, *P* = 0.236) and BAF (r = −0.059, *P* = 0.811).

**Table 3:** Patient Characteristics and Examination at T1

Characteristics	Patients (n = 19)
Median consultation interval (IQR)	4.0 (4.0–6.0)
Median BCVA, logMAR (IQR)	0.0 (0.1–0.0)
Visible spots/dots on fundus, n (%)	6 (31.58)
Clinical inflammatory signs, n (%)	
0	17 (89.5)
1	0 (0.0)
2	0 (0.0)
3	0 (0.0)
No data	2 (10.5)
Multimodal imaging inflammatory signs, n (%)	
0	17 (89.5)
1	2 (10.5)
2	0 (0.0)
3	0 (0.0)

BCVA = best-corrected visual acuity; IQR = interquartile range; logMAR = logarithm of the minimum angle of resolution.

### Multivariate Analysis

None of the predictors among sex (women, coefficient = 5.02, *P* = 0.765), age (by 1 year of age, coefficient = 0.89, *P* = 0.533), and BCVA (≥0.2 logMAR, coefficient = 0.35, *P* = 0.922) had a significant independent effect on ICGA. Similarly, neither sex (women, coefficient = 9.91, *P* = 0.510), age (by 1 year, coefficient = 0.77, *P* = 0.391), nor BCVA (≥0.2 logMAR, coefficient = −0.43, *P* = 0.961) were associated with significant changes in BAF.

## Discussion

In this study, we demonstrated that the total area and macular area affected by MEWDS lesions were significantly larger on ICGA than on BAF. This result corroborates the current hypothesis that the initial site of inflammation is the RPE, responsible for secondary and reversible damage to the outer segment of the photoreceptors (primary damage of RPE as pathogenesis of the lesions). In 1998, an in vivo study on monkeys demonstrated for the first time that the accumulation of ICG within the RPE was responsible for the late fluorescence observed in ICGA sequences.^22^ Subsequently, extensive experimental data from studies on both humans and animals have confirmed that the RPE progressively internalizes ICG following intravenous injection.^23^, ^24^, ^25^ Based on this postulate, Gaudric and Mrejen concluded that the hypofluorescent lesions on late-phase ICGA that do not show on early-phase ICGA were due to a defect in ICG internalization by the RPE by various mechanisms: this concerns acute syphilitic posterior placoid chorioretinopathy, choroidal hemangioma, drusen, and MEWDS. This should be distinguished from lesions of the choriocapillaris, which are hypofluorescent at all times in ICGA, such as acute macular posterior placoid epitheliopathy.^15^

Consistent with this, Zicarelli et al^14^ observed the development, in one of their patients, of new spots 48 hours after injection of indocyanine green, without any other injection in the interval. The fact that new lesions appeared at a distance from the injection reinforces the idea that the late hypofluorescence on ICGA comes from the RPE rather than the choriocapillaris. Indeed, hypofluorescence from the choriocapillaris is immediate and does not change over time. They also demonstrated hypofluorescent lesions in near infrared autofluorescence, supplanting the dark dots. As near infrared autofluorescence was strongly correlated with the level of melanin in the cells, they hypothesized that the photoreceptor damage was associated with a dysfunction of the RPE leading to a rearrangement of its melanosomes.^14^

In our study, we showed that all our patients had a spontaneously favorable evolution with an ad integrum restoration of their VA at the end of the follow-up. We did not find any correlation between the extent of macular damage on either ICGA or BAF and the recovery of VA. This supports the fact that inflammatory damage in MEWDS causes reversible damage to the RPE and also to the photoreceptors, without body cell loss. Onishi et al used adaptive optics scanning laser ophthalmoscopy to analyze the outer retina in 6 patients with MEWDS and found isolated damage to the outer segment of the photoreceptors without involvement of their inner articles or bodies. In their opinion, the fact that photoreceptors can still be identified at the site of certain lesions, thanks to adaptive optics scanning laser ophthalmoscopy, suggests that the RPE is a possible site of accumulation of subclinical material, without disturbing the overlying photoreceptors.^20^ This also appears to be the case in our study by analyzing the SD-OCT and en face OCT. Moreover, this form of damage, which involves the photopigmentary part of the cell and spares the photoreceptor nuclei, is the hallmark of retinal diseases that mainly affect the RPE and secondarily damage the photoreceptors.^26^, ^27^ This is the case, for example, with age-related macular degeneration. An impairment of the RPE, primarily because of decreased phagocytic and autophagic function, results in the accumulation of cellular debris. This progressive toxic overload eventually disrupts the normal functioning of the outer articles of the photoreceptors, leading to their degeneration and the premature aging of the retina.^26^

In a study of 36 eyes, Pichi et al^10^ found that the hyperautofluorescent lesions in BAF colocalized perfectly with the hyporeflective spots on en face OCT at the level of the ellipsoid, leading them to conclude that the primum movens was damage to the photoreceptors. The results of our study point in the same direction, although another interpretation is possible; in fact, the spots located in the ellipsoid zone are overlaid on the lesions in BAF but are smaller than the RPE lesions seen on late-phase ICGA. Therefore, MEWDS may cause higher damage to the RPE than to the photoreceptors. This constitutes an additional argument for initial damage to the RPE.

This study has several limitations, mainly related to its retrospective design. Multimodal imaging was incomplete for a proportion of patients. To increase the impact of our study, we therefore decided to select a smaller cohort with good-quality images for a more accurate analysis. Moreover, only a small set of patients underwent en face SD-OCT imaging, preventing wider analyses on this technique. However, this cohort is one of the largest identified in studies of the pathophysiology of MEWDS. A second limitation is that we were unable to computerize the area measurements because the gray levels of the BAF and ICGA images are not sufficiently contrasted for this. To limit this measurement bias as much as possible, we produced a standardized protocol so that the BAF and ICGA images were identical in terms of size and surface area to be studied. A single person (V.L.) carried out all the measurements for better reproducibility. Lastly, fundus fluorescence analysis can be hazardous, as it depends on a number of parameters, including the time of illumination with the excitation wavelength, lens opacity, or the patient's ability to fixate. For this reason, we have excluded patients with poor image quality or when fundus visualization was incomplete. We also had a rigorous method for calculating areas, as described previously. This allowed the same area to be analyzed for BAF and ICGA images of the same patient, eliminating, as far as possible, the imaging bias introduced by the variability of autofluorescence.

In conclusion, our study showed that MEWDS lesions are more extensive in ICGA than in BAF, indicating a predominant dysfunction in the RPE. We, therefore, support the hypothesis that MEWDS is a primary retinal pigment epitheliopathy, causing the reversible and nondestructive dysfunction of the RPE. Hyperautofluorescence could be explained by the loss of the outer segments of the photoreceptors that unmask the normal fluorescence of the RPE. Although it is not possible to exclude with certainty the primary involvement of the photoreceptors, our results support recent analysis of modern multimodal imaging. It would be interesting to carry out further studies to analyze the outer retinal layers in detail, in particular, using en face OCT to corroborate this hypothesis.
